# Retraction Note: Predicting cancer prognosis and drug response from the tumor microbiome

**DOI:** 10.1038/s41467-025-64170-y

**Published:** 2025-09-17

**Authors:** Leandro C. Hermida, E. Michael Gertz, Eytan Ruppin

**Affiliations:** 1 Cancer Data Science Laboratory (CDSL), National Cancer Institute (NCI), National Institutes of Health (NIH), Bethesda, MD USA; 2 Department of Computer Science, University of Maryland, College Park, MD USA

Retraction to: *Nature Communications* 10.1038/s41467-022-30512-3, published online 24 May 2022

The authors have retracted this article. Following the retraction of the original manuscript^1^ from which starting input for data processing was downloaded, the authors re-analysed their data with an updated computational pipeline but were unable to reproduce the original results. All authors agree to this retraction.
